# Additional Value of Peer Informants in Psychological Autopsy Studies of Youth Suicides

**DOI:** 10.1177/10497323211022316

**Published:** 2021-06-24

**Authors:** Milou Looijmans, Diana van Bergen, Renske Gilissen, Arne Popma, Elias Balt, Daan Creemers, Lieke van Domburgh, Wico Mulder, Sanne Rasing, Saskia Mérelle

**Affiliations:** 1 113 Suicide Prevention, Amsterdam, The Netherlands; 2 University of Groningen, Groningen, The Netherlands; 3 Amsterdam UMC, Amsterdam, The Netherlands; 4 GGZ Oost Brabant, Boekel, The Netherlands; 5 Radboud University, Nijmegen, The Netherlands; 6 Pluryn, Nijmegen, The Netherlands; 7 Dutch Center for Youth Health, Utrecht, The Netherlands

**Keywords:** suicide, adolescents, youth, young adults, mental health and illness, methodology, prevention, illness and disease, qualitative, psychological autopsy, the Netherlands

## Abstract

In this study, we examined the feasibility and added value of including peer informants in a psychological autopsy study of youth suicides. Peer semi-structured interview data from 16 cases were analyzed qualitatively and compared to parent data. Results show that peers added information to parents’ narratives in general and particularly on social relationships, bullying, school experiences, social media, and family relations. Peers also provided additional information on the presence of certain issues (such as social media contagion) as well as on the emotional impact from certain adverse events that seemed to have functioned as precipitating factors. We conclude that including peers in psychological autopsy studies of youth suicides is feasible and of added value but that more research is desirable. The results initially can be used in the design of psychological autopsies so that the maximum amount of information about each suicide will be learned.

## Introduction

Worldwide, suicide is the second leading cause of death among adolescents. Before the start of puberty, suicide is rare, but the incidence of suicide increases during the late teenage years (World Health Organization [WHO], 2014). Regarding prevalence, there has been an increasing trend in the number of suicides among 15- to 19-year-olds in the United States and the United Kingdom between 2000 and 2017 (Bould et al., 2019; Miron et al., 2019). In the Netherlands, which is the research site for the present study, the rates were relatively stable among 10- to 19-year-olds in the past years, with approximately 50 suicides (2.3 per 100,000 inhabitants) each year (Roh et al., 2018), although in 2017, there was a sharp and sudden increase to 81 (4.0 per 100,000 inhabitants; Centraal Bureau voor de Statistiek [CBS], 2018), a decline in 2018 to 51 (2.5 per 100,000 inhabitants; CBS, 2019), and a rise to 67 (3.4 per 100,000 inhabitants) in 2019 (CBS, 2020).

Risk factors for suicides among young people have been studied extensively (Bilsen, 2018; Cha et al., 2018; Franklin et al., 2017). The most important risk factors described in adolescents are mental disorders, previous suicide attempts, specific personality characteristics, genetic loading, and family processes, in combination with triggering psychosocial stressors (particularly victimization), exposure to inspiring models, and availability of (lethal) methods (Bilsen, 2018; Cha et al., 2018). Findings indicate an increased risk of suicidal ideation and/or suicide attempts associated with bullying behavior and cyberbullying (Klomek et al., 2010) and with social pressure and perfectionism (Donaldson et al., 2000; Mueller & Abrutyn, 2016). Furthermore, adolescents are vulnerable to risky behaviors because they are, on average, less able to regulate their impulses and look past the consequences of their behaviors (Sanci et al., 2018). Studies on adolescents show that binge drinking and substance abuse are associated with suicide and suicidal behavior (Miller et al., 2007; Schilling et al., 2009). As young people progress through adolescence, they also can be at greater risk of media exposure to suicide (Gould et al., 2003) or exposure to suicidal behavior in their general environment, which can create a contagion effect (Swanson & Colman, 2013). In addition, during adolescence, social media and contagious effects (Marchant et al., 2017) become a growing influence, and adolescents, besides experiencing positive aspects from social media, also can be exposed to cyberbullying (Brandau & Evanson, 2018) and sexting, which are known to provoke suicidal behavior (Medrano et al., 2018; O’Keeffe et al., 2011; O’Reilly et al., 2018). Finally, sexual and gender identity are relevant factors (Creighton et al., 2019), as lesbian, gay, bisexual, transgender, and queer (LGBTQ) youths are 4 to 5 times more likely to attempt suicide and 2 times more likely to think of suicide than heterosexual and gender-conforming youths (DiStefano, 2008; Russell & Fish, 2016; Salway & Gesink, 2018).

Despite research on risk factors for suicide in adolescence, more knowledge about the complex interaction among different risk factors is needed to better understand youth suicides (Bilsen, 2018). Psychological autopsy studies are viewed as one of the best methods for studying interactions among relevant factors leading up to a suicide (Cavanagh et al., 2003). In addition, these studies, in which participants talk about the lives of their deceased loved ones, can also be of great added value to relatives (Cooper, 1999). By conducting psychological autopsy studies and interviewing the people closest to the deceased, researchers can gain insight into the background of youth suicides, potential critical factors, and impactful situations and events related to the suicide (Cavanagh et al., 2003; Isometsä, 2001; Scott et al., 2006). While memory bias may play a role when informants narrate about the past, research has shown that such bias is less likely to occur for strongly negative life events (Assink, 2008), which are often associated with suicides. As the main subject in psychological autopsy studies cannot be interviewed directly, an important methodological challenge in psychological autopsy studies is how to arrive at sufficient reliable information about suicide. Two or more informants (interviewees) usually come up with more information about the background, characteristics, and precipitating factors of suicide than only one informant can. Therefore, to increase psychological autopsy studies’ reliability, it is best to include more than one informant for each suicide case (Brent et al., 1988). In most youth psychological autopsy studies to date, parents or other significant adults were included as informants, and many studies also included, yet not specifically analyzed, the independent contribution of interviews with peers (friends, siblings, other relatives, or neighbors who are about the same age as the deceased) (Brent et al., 1988; Houston et al., 2001; Lin et al., 2016; Portzky et al., 2005; Shaffer et al., 1996; Shafii et al., 1985).

The actual unique contribution of peers in psychological autopsy studies rarely has been investigated specifically. This may be due to practical and ethical issues. For example, including extra participants usually requires more financial resources, and interviewing minors (especially younger than 18) about the deaths of theirs siblings or friends requires even more careful preparation and very sensitive interviewing skills. Nevertheless, findings from empirical work point in the direction of peers’ unique role as potentially important sources of information for youth psychological autopsy studies for at least two reasons. First, the literature shows that when it comes to their children’s suicidal thoughts, there is a high prevalence of parental unawareness (Jones et al., 2019). Second, at the start of puberty, adolescents distance themselves from their parents while orienting more and more toward their peers in terms of their social relationships and social identity formation (Fuligni & Eccles, 1993), and around puberty, teenagers are more satisfied with their friendships than with any other relationships they have (Buhrmester & Furman, 1987).

It is theoretically and empirically plausible that peers (i.e., friends, siblings, and other relatives who are about the same age as the deceased) are of additional value in youth psychological autopsy studies, yet they are not systematically included in these types of studies. Our study aims to gather qualitative data related to the strengths and limits of including peers in psychological autopsy studies, particularly the added value of their data to inform future methodology. We expect this study to fill empirical and methodological gaps in the literature by examining the added value of including peers, in addition to parents’ contribution in a qualitative psychological autopsy of adolescent suicides as well as examining the feasibility of including peer informants. Therefore, our findings will be instructive for choices regarding the selection of informants in future psychological autopsy studies.

## Method

### Ethics

The Medical Research Ethics Committee of Amsterdam UMC approved the study (registration number: 2018.651—NL68348.029.18), and all participants provided their written informed consent. For minors (below the age of 16), parents provided written informed consent as well.

The interviews were always conducted in pairs, a senior interviewer who had the lead in the interview, who was accompanied by a junior interviewer. The pool consisted of four senior interviewers and three junior interviewers in total. All interviewers received training in the psychological autopsy method from one of the senior interviewers who is experienced in conducting qualitative interviews about suicidality with vulnerable people. Learning how to recognize complex grief among survivors was part of the training. Prior to their enrollment, it was examined whether survivors experienced suicidal thoughts above the cutoff point of the Suicidal Ideation Attributes Scale (SIDAS), in which case they would have been referred to care (and not enrolled in the study). The SIDAS is a validated measure to investigate current suicidal ideation (van Spijker et al., 2014). None of the interview candidates scored above the cutoff point on the SIDAS. Participants were contacted by telephone about a week after the interview to enquire about their well-being. Participants stated they found that the interview had been worthwhile and appreciated that they could discuss their beloved child, sibling, or friend and their lives in depth. Yet, some participants mentioned the interview had asked a lot from them emotionally and physically.

### Participants and Procedure

This study is part of a multimethod, psychological autopsy study of adolescent suicides conducted in the Netherlands (Mérelle et al., 2020). The Dutch Ministry of Health requested the research team to conduct a study into all 81 youth suicides (i.e., of youth below the age of 20) in 2017, as the number of youth suicides had peaked in this year. Nevertheless, the research team was aware that researchers in psychology autopsy studies can generally include only 25% to 60% of all cases in a certain time period. We therefore also worked with a saturation criteria, with 12 cases (Guest et al., 2006) being a minimum number to be included for saturation to occur. For the present study we thus considered 12 cases where peers had been interviewed next to parent to be a minimum as a comparison group to cases where peers could not be included.

For the broader research project, semi-structured interviews were performed with 54 parents, 19 peers, 11 teachers, and 11 healthcare professionals related to 35 young people (12 in the age group 14–16 years and 23 in the age group 17–19 years old) who died from suicide in 2017. Interviews were conducted from February to October 2019; thus, participants narrated about a suicide that happened on average 1.5 years ago. The participating peers were all suggested by the decedents’ parents who always functioned as first informants. An adult support figure was often, and always when a peer was younger than 16, present during the interviews. In some cases (i.e., with siblings), this support figure was a parent who also participated in the study. In 16 of the 35 cases, it was possible to interview both parents and peers. In this current study, only data from these 16 cases were analyzed.

### Recruitment

The recruitment of parents was conducted in four steps: (a) Coroners were kindly requested to identify 10- to 19-year-old youth who died by suicide in their regions in 2017, together with the contact details of general practitioners (GPs). (b) Coroners sent letters to GPs requesting them to inform the parents of youth who died by suicide about the study. (c) The parents who were interested were contacted by an interview coordinator. (d) Parents were also recruited through social media and traditional media, being respectful in terminology and mindful of media guidelines regarding reporting about suicidality.

### Inclusion of Peers

The Medical Ethical Committee of Amsterdam UMC that evaluated this study recognized the importance of involving peer informants, including peers aged below 16, and gave us permission to approach them through the parents of the deceased young people. Parents often felt reluctant to approach a peer because of the difficult topic. As a result, we were unable to speak to a peer in every case in our psychological autopsy study. Parents from 21 (of the 35) cases provided us with details on peers to ask to participate in the interview. No contact could be made with four of these peers, and one peer decided, after careful consideration, not to cooperate. Eighteen peers from the 16 remaining cases ultimately were interviewed of whom one peer was younger than 16 years old.

### Measures

The study topics for the semi-structured interviews were based on previous psychological autopsy studies conducted in Ireland, Belgium, Norway, and the United Kingdom (Arensman et al., 2016; Freuchen et al., 2012; Houston et al., 2001; Portzky et al., 2009; Rasmussen et al., 2014), and they were supplemented with questions from suicide experts, for example, “Did parents/peers noted a visible change in the behavior of young people shortly before young people had died by suicide?.” The interview topic list started with an open narrative part (Wengraf, 2001) in which informants responded to a broad open question about what had induced the development of suicidal behavior in the young person, followed by semi-structured questions about preidentified topics (adolescence, youth health care, clusters, contagion effects and social media, sexual and gender diversity, and ethnocultural and migration factors). Reflexivity during interviews, analysis, and writing was maintained by discussing and challenging assumptions among all researchers. Directly after each interview, each interviewer independently wrote a reflective report of one to two pages of each interview, zooming in on key events and important factors in the life and events related to the suicide of young people. All detailed information about the method for this psychological autopsy study is described by Mérelle et al. (2020).

### Analysis

All interviews were recorded digitally and transcribed verbatim. Data were coded and analyzed based on the constant comparative method (CCM, Boeije, 2002), which is one component of the grounded theory approach (Straus & Corbin, 1994). We worked with a version of CCM where we combined deductive and inductive analyses of data. Based on the interview instrument, we choose to first analyze data from the first broad, open question in the interview to see whether there were any differences in trigger factors mentioned first by parents and peers (a deductive approach). The open, narrative, pointed question was: “Looking at the life course of (name), can you tell me what you think has played a role in the suicide?” Second, we analyzed the interviews inductively, that is, we zoomed in on the contents of factors and circumstances listed as relevant to the suicide by the parent and peer informants. This resulted in the following list of topics: substance use; school experiences; social relationships with peers and bullying; family relations; social media and contagion effects; sexual and gender diversity; and suicide-related communication. These topics were a selection of all the topics that had been included systematically through a semi-structured approach in each interview. Topics we included were hence only selected for our study if they were seen as relevant by either parents or peers, and we subsequently examined their relative weight and detailed contents across each parent-peer dyad.

The current study views information provided by peers as “added value” when this information provided more or other meaningful insights into the mental health and circumstances of the deceased than the information that parents provided. Two researchers rated all data separately. When these two researchers rated information differently, the section was discussed with each other plus two other researchers. Through discussion, in which each researcher explained his own process of rating, unanimous agreement was reached in all cases on whether a section was of added value.

Analysis took place in two steps:

Step 1: Two researchers independently compared data from parents with data from peers regarding the aforementioned topics. The transcripts were compared to identify similarities and differences between these topics. All differences (i.e., more or other information was in the peers’ transcripts than in the parents’ transcripts) were coded. At this point, it was not yet examined whether this extra information was viewed as meaningful for understanding such suicides. During coding, we allowed the meaning of discrete text portions (the text as coded for each specific theme in the broader autopsy study) to be understood within the context of the full interview transcripts.
Step 2: Two researchers then categorized all coded data independently as follows:
  - The peer did not provide more or other information than the parent(s) did.
  - The peer provided more or other information than the parent(s) did about the same topic, but this information was categorized as not meaningful (details such as a peer revealing that the room the deceased slept in had a blue wall).
  - The peer provided more or different information than the parent(s) about the same topic, and this information was categorized as meaningful for understanding the suicide, that is, added value.

Finally, we compared the added information between friends and siblings to see whether any differences existed between them. For confidentiality reasons, it is not mentioned in the results whether data concerned a sibling or a friend.

## Results

The sample of siblings (8) and friends (10) consisted of 10 girls and 4 boys whom we interviewed individually, a boy and a girl who were interviewed together, as well as two boys who were interviewed together. Their ages ranged from 14 to 25 (*M* = 20.5; *SD* 3.1) All peers whom we interviewed indicated that they found the interviews to be pleasant as well as valuable in being able to (re)tell their stories to an objective listener:

The sincere interest in <name> does justice to him as a person

None of the peers reported any negative mental effects after the interviews or a need for any form of follow-up care, but four of the peers mentioned that they were tired physically after the interviews.

### Added Value Per Peer

All peers provided some form of additional information, except for one who did not provide additional information on any topic. The spread on the number of topics in which peers provided additional information varied from zero to seven. Ten peers provided additional information on at least four topics. There were no differences between siblings and friends, with one exception: siblings provided more information on family relations than friends did.

### Results Per Topic

**Figure 1.**
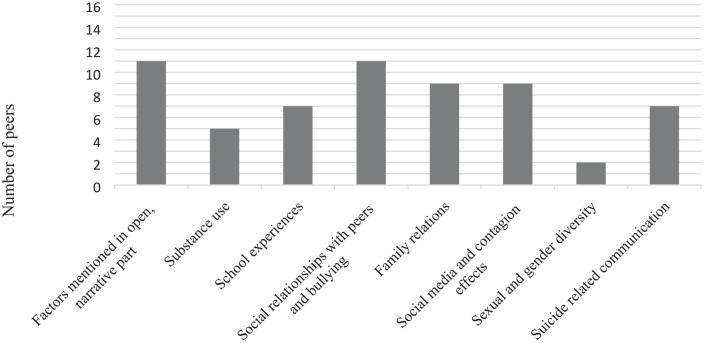
Number of peers who added information per topic.

In this section, we will describe the differences in factors mentioned with the first, open, narrative pointed question in the interview. Afterward, we will describe the most important results for each topic (substance use; school experiences; social relationships with peers and bullying; family relations; social media and contagion effects; sexual and gender diversity; and suicide-related communication). Figure 1 shows how many peers provided additional (i.e., new or different) meaningful information for each topic. Table 1 shows for which (sub)topics peers provided relevant additional data.

**Table 1.** Added Value by Peers Per Topic.

Topic	Subtopics Mentioned by Peers That Parents Did Not Mention in the Accompanying Cases	Number of Cases (*n*)
Substance use	Amount of alcohol/drugs	2
	Suspected of dealing soft drugs	1
	Used hard drugs for a while	1
	Sex in exchange for drugs	1
School experiences	Academic stress/problems	6
	Self-harm in class	3
	Unpleasant events or incidents	2
	Study doubts	2
	Very annoyed by classmates	1
	Overestimated at school because of strong social emotional skills	1
Social relationships with peers and bullying	Struggles with friendships	4
	Details about friendships and interactions	3
	Number of unhealthy friends increased	2
	Felt bullied for a while	1
	Never bullied while parents suspected bullying	1
	Parents’ aversion to friends pushed him toward friends	1
Family relations	Relationship with siblings	4
	Feeling guilt or difficulties for causing other people to worry	3
	Problems with parents and new partner	2
	Relationship/arguments with parents	2
	Worrying about other family members	1
	Reason parents divorced	1
	Influence of parental depression on a young person	1
	Abuse of parents by a young person	1
Social media and contagion effects	Social relationships in clinic	2
	Engaged in positive recovery communities	2
	More active on social media than parents thought	2
	Recontacted a parent via Facebook	1
	Removed the last name on social media and switched phone numbers often	1
	Suspected of blackmailing/threats via Facebook	1
	Encountering images of self-harm	1
	Very upset with a good friend’s suicide	1
Sexual and gender diversity	Contact with the one whom she had romantic feelings for was broken off	1
	Suspected difficulties with sexual identity confirmed by peer	1
Suicide-related communication toward parents/peers	Signals other than those parents received	6
	Showed self-harm only to peers	1

#### Differences in factors mentioned with the first, open, narrative-pointed question

We compared parents’ and peers’ answers to see whether differences existed between the factors that came to mind first when we asked the question: “Looking at the course of life of [name], can you tell me what you think has played a role in the suicide?”

In six cases, parents and peers provided different answers during the open narrative part. For example, in one case, parents mentioned attention-deficit disorder and inadequate health care as important factors, while the peer mentioned psychoses, a tense home environment, and drug use. In another case, the parents mentioned specific setbacks in the last period before suicide, while the peer said that fear of failure and getting angry with himself were important factors.

In 10 cases, parents and peers reported similar factors, but of these 10 cases, there were 2 in which parents mentioned more additional factors and 6 cases in which peers mentioned more additional factors. For example, in one case, parents and the peer mentioned autism as an important factor in the suicide, but the peer added information about precipitating factors, such as that the young woman had a hard time with a relational breakup and how her scooter broke down during the month before her suicide.

Altogether, parents were more inclined than peers to use a terminology of psychiatry diagnoses and suboptimal mental healthcare services as relevant to suicide. Possibly, this may suggest that parents may be more apt to point to the biomedical and healthcare model to understand the suicide of the young person compared to peers.

#### Substance use

Peers made relevant additions in five adolescent suicide cases on the topic of substance use. The parents in one case said they were aware that their child used (hard and/or soft) drugs or alcohol, but peers provided more details concerning how much and how often alcohol or drugs were used. One peer added that his friend was under the influence of drugs in class without teachers noticing or parents knowing. Another peer added that certain WhatsApp messages showed that one deceased adolescent also may have been dealing marijuana, which the parents did not mention. Another peer added that the youth used hard drugs for a while but that he stopped using after a stern conversation with his father, although his parents did not mention this. Finally, in one case, the parents told us that their daughter was engaged in sexual solicitation, and a peer explicitly said that she was having sex in exchange for drugs.

Taken together, peers seemed to be more knowledgeable about drug and alcohol behavior of the young person than parents. Sometimes, peers had been present when the substance abuse occurred as a witness and/or a compliant. In addition, some peers may have known more than their parents, since youth did not want to upset or disgrace their parents through their substance abuse.

#### School experiences

In seven cases, peers provided additional content regarding school experiences that parents did not mention at all or not in the same way. Most were about disappointments for the adolescents, such as a certain school grade not working out or that they were allocated by school management to a lower level; doubts about the course of study they had chosen; and not being able to be proud of their academic performance. Parents talked about these events (such as the transition to a lower school level), but peers’ additional content made the negative impact on the adolescent more clear and highlighted how the young person had been more concerned with certain school-related events than parents seem to have suspected.

Some young people who had died by suicide experienced lots of pressure and stress at school. For example, one parent said her child always was very punctual with her schoolwork, set high academic standards, and wanted to do everything very well. The peer’s information added that this academic pressure led to crying spells at school and self-harm to punish herself if something went wrong related to the school context. In one case, the youth’s class was described as “a bit of a sickly class where a lot of self-harm took place,” while the parents did not say anything about this in their interview. Also, two peers talked about unpleasant events or incidents at school (such as getting tied up by a teacher in a special education school or getting laughed at while giving a presentation). One peer told us that her sister could get very annoyed with his classmates, and another peer thought his friends were overestimated in school level because of their strong social–emotional skills.

Altogether, peers may have a more detailed understanding of stressful school events faced by the young person than parents, particularly when these peers went to the same school as the young person and were firsthand witnesses of these events.

#### Social relationships with peers and bullying

In 11 cases, peers provided additional information on social relationships and bullying. The peers elaborated on what the parents had said. For example, one peer revealed more about how the youth specifically felt in her group of friends, how much pressure she felt to be liked by her peers, and that her mother’s aversion to the group of friends probably had the opposite effect on the deceased young person. In three cases, peers provided more than parents did, including information on how the deceased interacted with friends and what kind of conversations they had.

In one case, parents indicated that their daughter had less time for her old friends because of her new school and due to her enthusiasm for new friends. However, the peer told us that the reduced contact with her old friends was very difficult for her and that she was very uncertain about her new friendships and wondered whether she had enough “nice friends.”

Two peers indicated that what they termed one youth’s “sick” group of friends increased and that those whom they perceived as “healthy” friends no longer remained, which they felt also strengthened the youth’s “sick” side.

In one case, a peer said the deceased adolescent felt bullied for a while in secondary school, although the youth’s parents simply answered “no” when they were asked whether their child had ever been bullied. By contrast, in another case, a parent suspected that something was going on at school and that her child might have been bullied, but according to the peer, in this case, the youth was never bullied.

#### Family relations

For nine cases, additions were made on factors related to family relationships. Several additions from peers had to do with feelings of guilt toward parents, including one youth who found it difficult when their parents worried about them:

Her parents were good to her, but I think [ . . . ] her mother was very, very worried. And then it was completely appropriate in this situation, but I think her mother has always worried about her disproportionately.

Two peers said the youth had many arguments with her parents. One of these peers explicitly said that the youth did not have a good relationship with her parents and felt very alone, while these parents mentioned in more general terms that contact with their child had become increasingly difficult since puberty. Furthermore, some other peers described some youths’ bonds with siblings in a different (but not contrasting) or more elaborate or extreme way than parents, for example, one peer indicated that one youth “hated her sister to the bone.”

Another peer said one youth started to assault her parents physically at some point, although the parents never mentioned this. Also, in two cases, there were problems in the relationship between the youth, the youth’s father, and the father’s new partner during childhood. The parents mentioned this, but peers provided more details about what these problems entailed and how they impacted the youth.

Finally, in one case, the parents reported that in retrospect, one parent’s depression may have impacted the youth more than they thought, but the peer provided an extensive description of how challenging and impactful the parent’s mental illness probably was for the deceased young person:

And she always felt that tension immediately. [ . . . ] Then she started to soften that a bit. Or just saying the right words so that it didn’t escalate. Not that it ever escalated here, but she did have that antenna for that tension.

Thus, young people may have confided in their peers somewhat more when they were feeling unhappy over their relationship with their parents. Possibly, young people perceived peers as a more suitable party to confide in and seek social support from, as peers were themselves uninvolved in these arguments or tensions with the mother or father. Alternatively, parents may have found it too painful to narrate in depth about tensions in the parent–child relationship.

#### Social media and contagion effects

Peers provided additional information on social media and contagion effects in nine cases. Peers often knew a little better than parents which social media platforms a youth used. Two times, parents called their child “not so active” on social media, while peers stated that they were actively involved with it. One peer said that a few years before a youth’s death, she contacted her mother, with whom she had not communicated with for years, via Facebook. The father did not mention this at all during the interview.

Another peer said youth at some point removed his last name on social media, often obtained a new phone number, and used Facebook Messenger instead of WhatsApp, which she found remarkable. The same peer also said that he once saw something on this youth’s Facebook page that raised suspicions over “lover boys” (i.e., “Romeo pimps”) or at least threats from men related to romantic or sexual relationships. Another peer said the youth looked at many images of self-injury and scars on Tumblr and triggered himself with this. Two other peers indicated that the youth consciously avoided triggers for self-harm on social media, engaged in positive recovery communities, and that peers who also self-harmed or attempted suicide were positive and helping contacts. On the other hand, another peer said that while she generally believed communities of peers with lived experience with self-harm can be helpful, in the case of her deceased friend, there were risks involved regarding developing a new identity around self-harm. Some parents also mentioned this but not in this specific case.

Another peer said a youth had a friendship in a clinic that was very “toxic,” for example, it included a ritual of sticking plasters on each other’s wounds after self-harm. Parents also viewed this friendship as destructive, but peers provided more details about this friendship. One peer who had stayed with her friend in a clinic provided more details than the parent(s) about what actually happened there regarding self-harm and that young people there hurt themselves competitively. One peer told us that the young person was very upset by a friend’s suicide; the parents mentioned the suicide but did not say it was a friend of their child’s.

Taken together, peers may be more knowledgeable than parents about the role of social media and contagion effects, since they were either also active in or a close witness to the same online social networks as the young person.

#### Sexual and gender diversity

There were two LGBTQ cases in which a peer provided additional information on this topic. In the first case, the mother had suspected, yet never asked, whether her child had some difficulties with her (gay) sexual identity, and the peer confirmed that this had been the case. In addition, in another LGBTQ case, a mother and peer both said that there had been a situation of unanswered love from a same-sex friend of youth, but the peer provided more details and noted that the friendship eventually ended against the youth’s will.

#### Suicide-related communication with parents and peers

In six cases in which several suicide attempts had been made, and the suicides were not unexpected, both peers and parents received multiple signals and knew the young person was suicidal, mainly because of previous suicide attempt(s) and their openness about their suicidal ideation. However, peers often received different signals than parents, such as a farewell message half an hour in advance, messages on social media, or the message that the deceased had been at the train station for a long time a few days before the suicide.

In a seventh case, the deceased often told his peer that he really was “done with it” when his mother threw him out of their home again and that the only way to find peace would be suicide. Another youth only showed and talked about her self-injuries to a peer, but her parents knew she was harming herself, but she did not want to talk to anyone else about it.

## Discussion

This study examined the added value and feasibility of including peers in a psychological autopsy study of 16 cases of youth suicides. Findings showed that peers added information to parents’ narratives on every topic but mostly on school experiences, social relationships, bullying, family relations, social media, and contagion effects. In general, peers were able to clarify certain statements and the impact on youth because they had been present as witness or compliant during the events or incident perceived as stressful for youth (e.g., bullying incidents, chats from “romeo pimps,” and alcohol and drug abuse), whereas parents were not present on those occasions.

In addition to peers having firsthand information as witnesses, we noted a number of situations and additional reasons why peers were valuable in adding more information to parents’ stories. Regarding family relations, siblings added more information on these youths who died from suicide, including more details than other peers (friends/relatives) provided. This was particularly the case for information related to relationships within the family and the atmosphere at home. Regarding child abuse, Moskos et al. (2005) showed that friends and nonparental relatives more often reported that the decedent had experienced emotional and verbal abuse, whereas parents more often reported physical and sexual abuse of the decedent (Moskos et al., 2005). We also found that peers added more information not so much about abuse but particularly about these youths’ difficult relationships and fights with their parent(s). Possibly, youth confide more in peers than parents when they face poor family relations, as they may prefer to seek social support from someone not involved in their arguments or tensed relationship with their parent. For research into risk factors and prevention strategies related to the home and family situation, it seems important to base information not only on stories from parents but also on those from peers, especially siblings.

Regarding social media and contagion effects, the results showed that peers often knew better than parents what type of social media platforms the decedents used actively and also provided more insight into social media’s contagion effects. Peer stories revealed that on one hand social media was helpful for youth, since parents and peers stated that online communities offered their child/ peer with an opportunity to provide online support to vulnerable peers, which they valued. However, sometimes peers in these online groups would post visual images or messages about self-harm which created potential contagion effects or were a burden on youth in our study. This result could be valuable when developing suicide prevention strategies for youths via social media.

Overall, peers specifically provided additional information about precipitating factors (Turecki et al., 2019), often including more details on certain adverse events’ emotional impact on these youths, such as bullying, school-related problems, relationship break-ups, and stress-related factors in the home context. This corresponds with studies showing that adolescents develop stronger emotional relationships with peers and that parents’ role in adolescents’ lives decrease during adolescence (Buhrmester & Furman, 1987; Fuligni & Eccles, 1993).

With regard to predisposing factors, peers in our study provided additional information about perfectionism, a personality trait associated with suicidal ideation and attempts (Smith et al., 2018). Thus, there were specific topics in which peers added meaningful information on top of parents’ narratives. From this, we could conclude that peers provided information on topics that parents did not know about, viewed as irrelevant to mention, or found too painful to talk about because they may have exposed less-favorable aspects about themselves or their children. Therefore, it seems important to supplement parents’ narratives with those of these youths’ peers. This corresponds with a study by Moskos et al. (2005) who concluded that parents and friends (including siblings, other relatives, teachers, and employers) were more sensitive in recognizing risk factors for suicide (Moskos et al., 2005).

### Feasibility

Overall, peers appreciated the qualitative interviews during which they could speak freely about the decedent’s life. Considering that parents in 14 (out of 35) cases did not submit any peers’ names to interview, we were able to interview only peers and compare the parents’ and peers’ stories in 16 cases. Parents’ reluctance to ask peers to participate in our study often originated from the assumption that it would be “too heavy” for young people to engage in interviews about deceased youths. However, peers who participated in our project found it valuable to do so and did not view it as harmful. This is a worthwhile result to communicate to parents and to healthcare professionals to encourage future enrollment of peers in psychological autopsy studies. All peers, except for one, added information to the parents’ narratives. The one peer who did not add anything on any topic was a friend of the deceased who was only 12 at the time of his friend’s suicide. Although this can be related to many factors other than age, such as personality, we think that one must reach a certain age to be able to retell a story and reflect on such a big life event. Further research could provide more insight into this but should consider that it is not always possible to interview a peer in every suicide case.

### Strengths and Limitations

We analyzed information provided by peers of different ages and with different relationships to the deceased. Also, due to the extensive semi-structured interviews, there was a lot of rich data on the lives of the deceased, so we could compare the information that parents and peers provided on many different topics.

The success rate of inclusion of parents in our psychological autopsy study (i.e., 45%) is in line with results from other research. Nevertheless, as we only targeted one year (2017), and the Netherlands is a relatively small country in terms of population size, this has resulted in a relatively small sample size.

We realize that when drawing conclusions, we must take into account the methodological and interpretive challenges of our narrative data (Bantjes & Swartz, 2019). Due to the semi-structured interview instrument, not all questions were asked in the same way in every case. Some participants told us things on their own accord that others also might have told us if they would have been asked specifically. Furthermore, seven peers had an older support figure present during the interview that might have influenced the peers’ openness. In three cases (when the peer was a sibling), this support figure was a parent who already had taken part in the interview themselves. In one case, a brother and sister were interviewed at the same time, which also may have influenced their answers. In addition, we interviewed the parents and peers, on average, 1.5 years after the youths’ suicides. In all cases, the peers and parents already had spoken to each other, so information had been exchanged about the youths’ lives, which could have led to a shared narrative and, therefore, bias. Furthermore, although all the peers had been close to the young person and showed in-depth understanding about their lives, they were recommended by the parents, which also could have led to shared perceptions and information bias. Nevertheless, for ethical and practical reasons, we argue that parents will need to be the first party to nominate a peer as an interview candidate in future research. Finally, adolescents with a migrant background were underrepresented in this study, which underpins the need of examining how migrant parents can be involved in psychological autopsy studies.

## Conclusion

Although including and interviewing young informants in a psychological autopsy study is ethically, economically, and practically challenging, these young informants provide critical new information on influential factors associated with youth suicides. Peers provided information into deceased youths’ lives that differed from those of parents, adding more insight into the suicidal process by providing more information about determinants, such as psychological and social factors, adverse events, social media, and family relations. As far as we know, this is the first qualitative study that systematically compared information from parents and peers from a psychological autopsy study in young people on so many different topics.

Thus, to learn the most from youth suicides by means of psychological autopsy, we recommend involving peers as participants. In addition, in terms of prevention, we recommend involving peers in the treatment and recovery of young people with mental health problems. Peers could receive psychosocial education about, for example, suicide warning signs and how to be helpful in supporting treatment adherence and positive behavioral choices. Subsequently, they potentially could become involved as part of a “Youth-Nominated Team Intervention,” which has proven to be associated with reduced mortality in adolescents (King et al., 2019).

## Supplemental Material

sj-pdf-1-qhr-10.1177_10497323211022316 – Supplemental material for Additional Value of Peer Informants in Psychological Autopsy Studies of Youth Suicides Click here for additional data file. Supplemental material, sj-pdf-1-qhr-10.1177_10497323211022316 for Additional Value of Peer Informants in Psychological Autopsy Studies of Youth Suicides by Milou Looijmans, Diana van Bergen, Renske Gilissen, Arne Popma, Elias Balt, Daan Creemers, Lieke van Domburgh, Wico Mulder, Sanne Rasing and Saskia Mérelle in Qualitative Health Research
